# Transcranial Sonography Characteristics of Cerebellar Neurodegenerative Ataxias

**DOI:** 10.3390/brainsci14040340

**Published:** 2024-03-30

**Authors:** Olivera Tamaš, Milija Mijajlović, Tamara Švabić, Milutin Kostić, Gorica Marić, Andona Milovanović, Marta Jeremić, Nataša Dragašević-Mišković

**Affiliations:** 1 Neurology Clinic, University Clinical Centre of Serbia, Faculty of Medicine, University of Belgrade, 11000 Belgrade, Serbia; milijamijajlovic@yahoo.com (M.M.); tasha.svabic@gmail.com (T.Š.); andona8@gmail.com (A.M.); marta.jeremic@gmail.com (M.J.); ntdragasevic@gmail.com (N.D.-M.); 2 Institute of Mental Health, Faculty of Medicine, University of Belgrade, 11000 Belgrade, Serbia; milutin.kostic@imh.org.rs; 3 Institute of Epidemiology, Faculty of Medicine, University of Belgrade, 11000 Belgrade, Serbia; goricamaric87@gmail.com

**Keywords:** neurodegenerative cerebellar ataxias, transcranial sonography, hyperechogenicity, ventricle enlargement, raphe discontinuity

## Abstract

Cerebellar neurodegenerative ataxias are a group of disorders affecting the cerebellum and its pathways with different neurological structures. Transcranial sonography (TCS) has been used for the evaluation of brain parenchymal structures in various diseases because of its fast and safe utilization, especially in neuropsychiatric and neurodegenerative diseases. The aim of our study was to investigate TCS characteristics of patients with neurodegenerative cerebellar ataxias. In our study, we included 74 patients with cerebellar degenerative ataxia; 36.5% had autosomal dominant onset, while 33.8% had sporadic onset. Standardized ultrasonographic planes were used for the identification of brain structures of interest. The SARA, INAS, neuropsychological and psychiatric scales were used for the further clinical evaluation of our study participants. The brainstem raphe was discontinued in 33.8% of the patients. The substantia nigra (SN) hyperechogenicity was identified in 79.7%. The third and fourth ventricle enlargement had 79.7% and 45.9% of patients, respectively. A positive and statistically significant correlation was found between SN hyperechogenicity with dystonia (*p* < 0.01), rigidity and dyskinesia (*p* < 0.05). The higher SARA total score is statistically significantly correlated with the larger diameter of the III (r = 0.373; *p* = 0.001) and IV ventricles (r = 0.324; *p* = 0.005). In such patients, the echogenicity of substantia nigra has been linked to extrapyramidal signs, and raphe discontinuity to depression. Furthermore, ataxia and its clinical subtypes have positively correlated with the IV ventricle diameter, indicating brain atrophy and brain mass reduction.

## 1. Introduction

Cerebellar neurodegenerative ataxias are a rare and diverse group of neurological disorders. The affection of the cerebellum as its main entity with its specific pathways is the reason for its main clinical features, which, in essence, is ataxia. Its clinical features can also affect speech, gait and limb coordination. Even though ataxia is the main clinical finding, and since neurodegenerative ataxias represent a diverse neurological disease group, their clinical manifestations may differ. For example, autosomal dominant cerebellar ataxias or spinocerebellar ataxias (SCAs) are characterized by cerebellar signs as well as additional non-cerebellar signs. Non-cerebellar signs can be very heterogenous and may include extrapyramidal features such as the presence of rigor and bradykinesia. Pyramidal signs can also be present with respect to spasticity and paresis. In some cases, signs of peripheral neuropathy can also be detected with nerve conduction studies. In some types of SCAs, cognitive deficits have also been observed [1,2]. SCA1 and SCA2 are cerebellar ataxias that are genetically determined and hereditary; these two types of cerebellar ataxias are caused by trinucleotide expansions. Reviewing the epidemiological data on neurodegenerative ataxias in Serbia, we note that SCA 1 and SCA2 are the most common types of hereditary ataxias in the Serbian population [3]. Friedreich’s ataxia (FRDA) is a progressive, multisystemic neurodegenerative disease clinically presenting with ataxia, cardiomyopathy and other cardiac complications as well as musculoskeletal deformities and endocrine disorders such as diabetes. FRDA is the most common autosomal recessive ataxia. The neurological clinical hallmark of FRDA is a progressive mixed cerebellar–sensory ataxia [4,5]. A separate group of neurodegenerative ataxias represent patients with clinical signs that correspond to hereditary neurodegenerative ataxias (cerebellar, extrapyramidal, pyramidal and other neurological features) but have a negative family history and are defined as a sporadic ataxia of unknown cause. However, recent research has shown that a number of them carry new mutations [6,7]. It is important to differentiate sporadic adult-onset ataxia (SAOA) from atypical parkinsonism forms such as multiple system atrophy (MSA-C), which may also represent some overlapping clinical features, as the prognosis and treatment are different for these two disease groups [8].

Transcranial sonography (TCS) has been used for over 20 years for the evaluation of brain parenchymal structures in various diseases, especially neuropsychiatric and neurodegenerative diseases. By visualizing different brain structures with TCS, it is possible to evaluate certain conditions such as hydrocephalus or the severity of brain atrophy. By identifying the echogenicity of certain structures, it is possible to detect disorders such as Parkinson’s disease, depression, dementia and other neurodegenerative processes. Over the years, it has found wide use due to its numerous qualities, which, in the first line, are low examination time, low price and high sensitivity and specificity, all while the whole diagnostic process is safe and does not include an admission of X rays [7,9]. The assessment of different brain structures, such as the evaluation of the echogenicity of substantia nigra (SN), has been used for the determination of the diagnosis of Parkinson’s disease and for monitoring disease progression in patients with Parkinson’s disease [8,10]. The visualization of brainstem raphe using the mesencephalic plane of insonation and the evaluations of its continuity have found great importance in the diagnosis of depression. In multiple studies evaluating TCS characteristics of patients with depression, it was found that in these patients, the brain raphe is discontinued or completely absent, enabling the diagnosis of depression to be established. The measurement of the brain ventricles (lateral, third and fourth) by TCS has found its clinical significance in the estimation of hydrocephalus and its progression, as well as in the estimation of cortical and cerebellar atrophy. One of the rare limitations of TCS is the inability to be conducted in all subjects due to untransparent temporal windows in some subjects [9,11]. Only a small number of published articles describe TCS characteristics of patients with neurodegenerative cerebellar ataxias [10,12]. Since TCS is a safe, fast and widely available technique, the evaluation of the TCS characteristics of patients with cerebellar neurodegenerative ataxias can be of both diagnostic and clinical significance. The aim of the present study was to investigate the TCS characteristics of patients with neurodegenerative cerebellar ataxias.

## 2. Materials and Methods

### 2.1. Study Design

Our study is a monocentric, retrospective, cross-sectional study. The study was conducted in the Neurology Clinic, Clinical Center of Serbia. The time window of the study was from 2017 to 2020. Patients were adults of both sexes, aged ≥18 years, who were examined at the study site. TCS was performed on 74 patients who had suitable, transparent temporal bone windows and had a confirmed diagnosis of neurodegenerative cerebellar ataxia. The examiner performing TCS was blinded during this study. 

### 2.2. Selection of Participants

In total, 74 patients with a confirmed diagnosis of neurodegenerative ataxia were obtained for the study. The diagnosis was obtained either by genetic analysis combined with clinical and neurological features or in case of sporadic cerebellar ataxias by clinical and neurological examinations and with other diagnostic means such as magnetic resonance imaging and other laboratory tests ruling out acquired forms of cerebellar ataxias (i.e., due to immune-mediated, toxic or metabolic etiologies). For hereditary forms of ataxia, family history and family trees were obtained in order to determine genetic relations and inheritance. From the total patients with neurodegenerative cerebellar ataxia, 10 patients were diagnosed with SCA1 and 6 with SCA2. One patient had an SCA 7 mutation. For 10 patients, no genetic mutation was identified that would determine a definite type of hereditary cerebellar ataxia; however, the family tree analysis and family history show a clear and apparent autosomal dominant pattern of inheritance. Such patients were described in our study as genetically undetermined but hereditary ataxias. A total of 15 patients had an autosomal recessive type of cerebellar ataxia. FRDA was identified in five of these 15 patients. An ANO10 mutation was present in seven of these 15 patients. Two patients were identified as having a positive mutation in the RFC1 gene. One patient was classified as genetically undetermined since the precise genetic mutation was not identified, but a clear autosomal recessive inheritance pattern was established due to the family tree and family history. Twenty-five patients had a diagnosis of sporadic neurodegenerative cerebellar ataxia (SAOA), and because of the dominant cerebellar clinical feature, seven patients with MSAc were also included in our study. 

Patients who already fulfilled the criteria for establishing a diagnosis of another neurodegenerative disease, which in its clinical presentation could cause ataxia, were also not included in this study. Patients who, due to other comorbidities, could not fully participate or fulfill all the study requirements, such as patients with major psychiatric disorders, were excluded from the study.

The participants enrolled in the study underwent clinical and neurological examination as well as other relevant diagnostic tests. Collected data also included information on age, gender, age at disease onset and disease duration.

In addition to the previously described patients with ataxias, 31 age and sex-matched control subjects (CS) were enrolled in the study. 

The study was approved by the Ethical Committee of the Faculty of Medicine, University of Belgrade, and all participants signed informed consent. Institutional Review Board Statement: The study was approved by the Ethical Committee of the Faculty of Medicine, University of Belgrade (1322/V-11, 10 March 2021). All participants signed informed consent.

### 2.3. Instruments

For the evaluation of clinical and neurological symptoms and signs in our study patients, several well-known scales were used in this study, which have been described in detail in our previous studies [13,14]. The severity of ataxia was assessed using the commonly used and validated scale: Scale for Assessment and Rating of Ataxia (SARA) [15]. Other instruments and scales used for the scoring and assessment of the severity of ataxia were the Spinocerebellar Ataxia Functional Index (SCAFI) [16] and the Composite Cerebellar Functional Severity Score (CCFS) [17]. Non-cerebellar signs were evaluated using the Inventory of Non-Ataxia Signs (INAS) [18]. Autonomic dysfunction was also evaluated in every patient in this study by using the Autonomic Symptoms Assessment Questionnaire (SCOPA AUT) [19].

Apart from ataxia, non-cerebellar symptoms and autonomic symptoms, the cognitive functions of our patients were also assessed. For that purpose, global cognitive function was evaluated using Addenbrooke’s scale for assessing cognitive function: Revised version (ACE-R) [20] and by Mini-Mental State Examination (MMSE) [21].

### 2.4. Assessment of Depression

Since psychiatric disorders are very common among neurodegenerative diseases, we have also incorporated such a segment in our study in regard to the assessment of depression in our patients. The Hamilton Depression Rating Scale (HAMD) was used in our study for the assessment of the presence of depression in our study subjects [22]. The testing consisted of a structured interview carried out by a trained physician. The testing was conducted in a one-by-one manner in a closed setting, with only the trained investigator conducting the interview and the study subject. 

While conducting the interpretation of the obtained results, a score of 0–7 on the HAMD scale was generally accepted to be within the normal range, while a score of 17 or higher was highly indicating at least a moderate severity of depression.

### 2.5. Transcranial Sonography

TCS was performed in the ultrasonography department of the Neurology clinic, Clinical Center of Serbia, using Esaote Technos MP, Italy. The ultrasonography machine is a color-coded phased-array ultrasound system equipped with a 2.5 MHz 90° sector probe. The examination was conducted through the preauricular acoustic bone windows. Before the beginning of the examination, every patient was evaluated to determine whether the temporal acoustic bone windows were transparent and whether the ultrasonographic image was interpretable. The following ultrasound parameters were set: penetration depth of 16 cm, dynamic range of 45 dB, high persistence and reject 7. The best quality of the image was obtained by simultaneously adjusting the image brightness, contrast and time-gain compensation. Patients who did not have adequate temporal bone window quality or had non-ultrasonographical transparent temporal windows were excluded from the study. A TCS examination was performed by an examiner who was certified in conducting TCS and had at least five years of experience in conducting this diagnostic method. Because of the need for precise identification of brain structures and their echogenicity, the ultrasonographer was a neurologist. The examiner conducting TCS during this investigation was blinded and was not informed of the patient’s diagnosis or any other clinical and neurological features of the patient. The examiner was not informed of the patient’s genetic testing results or their MRI findings. Standardized ultrasonographic planes were used for the identification of brain structures of interest, and the periauricular and transtemporal approach was used for the visualization of the brain’s structures. The TCS characteristic of SN was measured after its identification and localization within the mesencephalic brainstem. Echogenic size measurements of SN were evaluated using an axial TCS plane and on axial scans after manually encircling the outer circumference. The echogenic size of SN less than 0.19 cm^2^ was classified as normal in its echogenicity; echogenicity and size above 0.24 cm^2^ were defined as significantly hyperechogenic. The dimensions of the SN 0.19–0.24 cm^2^ were defined as moderately hyperechogenic. During the TCS evaluation of each patient, visualization and measurements of SN were performed on each side (both SN regions of the brain). For the classification of patients with respect to their SN echogenicity, the greater value of bilateral SN echogenic sizes of each individual was used. The sum of both echogenicities of SN was also used for further evaluation and interpretation. The echogenicity of the brainstem raphe was evaluated semi-quantitively as it is usually performed in clinical practice. Its echogenicity was compared to surrounding brain structures, which are usually the red nucleus (NR) and basal cisterns. In the case of normal echogenicity, raphe was isoechogenic in comparison to the NR and continuous in its structure. A hypoechogenic raphe was defined as discontinuous or completely absent and represented a pathological variant. Such results were also represented in numerical form, with 1 being a continuous raphe and 0 being a discontinuous or absent raphe. The widths of the third and fourth ventricles were measured on a standardized diencephalic axial scanning plane. The identification of two parallel TCS hyperechogenic lines representing the III ventricle and the longest diameter of distance represented the size of the III ventricle. A diameter larger or equal to 6mm was considered abnormal (enlarged). The diameter of the IV ventricle was assessed qualitatively. In the case of its clear visibility and in contrast to the echogenicity of adjacent brain structures, it was defined as enlarged. In case of poor or no visibility, it was defined as normal sized [11]. 

### 2.6. Statistical Analysis

Demographic and clinical characteristics of participants were presented using descriptive statistics. Continuous variables were described with mean and standard deviation, and nominal and categorical data were presented using frequencies and percentages. The relationship between continuous variables was assessed by the Pearson correlation coefficient, while in other cases, Spearman’s nonparametric correlation analysis was used. For the evaluation of frequencies, a chi-square test was used. For small samples, Fisher’s exact test was used. Continuous numeric variables were assessed using the student *T*-test. A *p*-value of less than 0.5 was considered significant. All analyses were performed in SPSS (Statistical Package for Social Sciences) version 20.0.

## 3. Results

Demographic and clinical data are shown in Table 1. In our study, there was a male predominance of patients (63.5%), and the mean age was 48.1 ± 12.9 years. The mean disease duration was 11.7 ± 9.6 years. The disease type that predominated was either dominant hereditary or sporadic. In Table 1. The SARA, ACE, MMSE and Hamilton depression scale scores are presented. The mean SARA score was 15.3 ± 6.8; for the INAS, it was 4.5 ± 2.0; for SCOPA AUT, it was 5.1 ± 6.8; and for ACE-R, it was 77.7 ± 17.2. 

**Figure 1:**
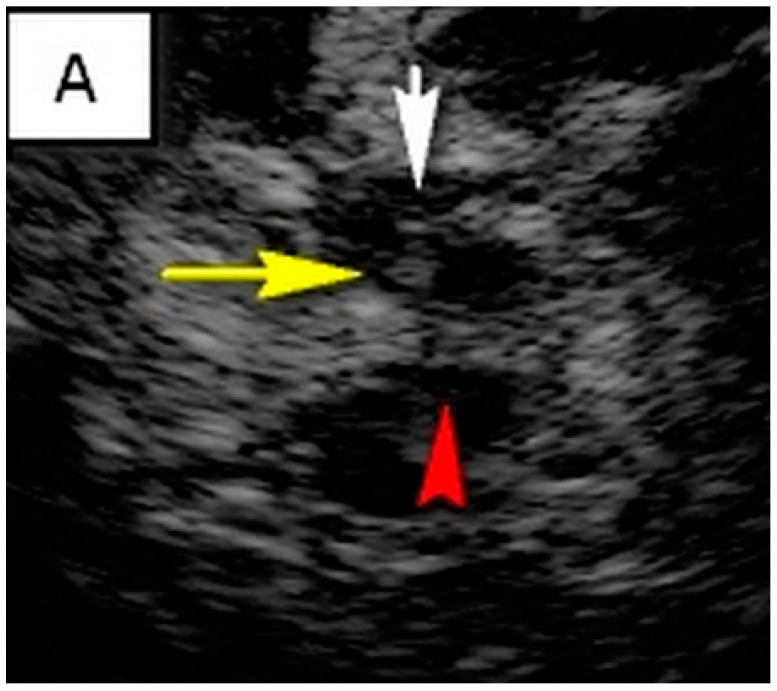

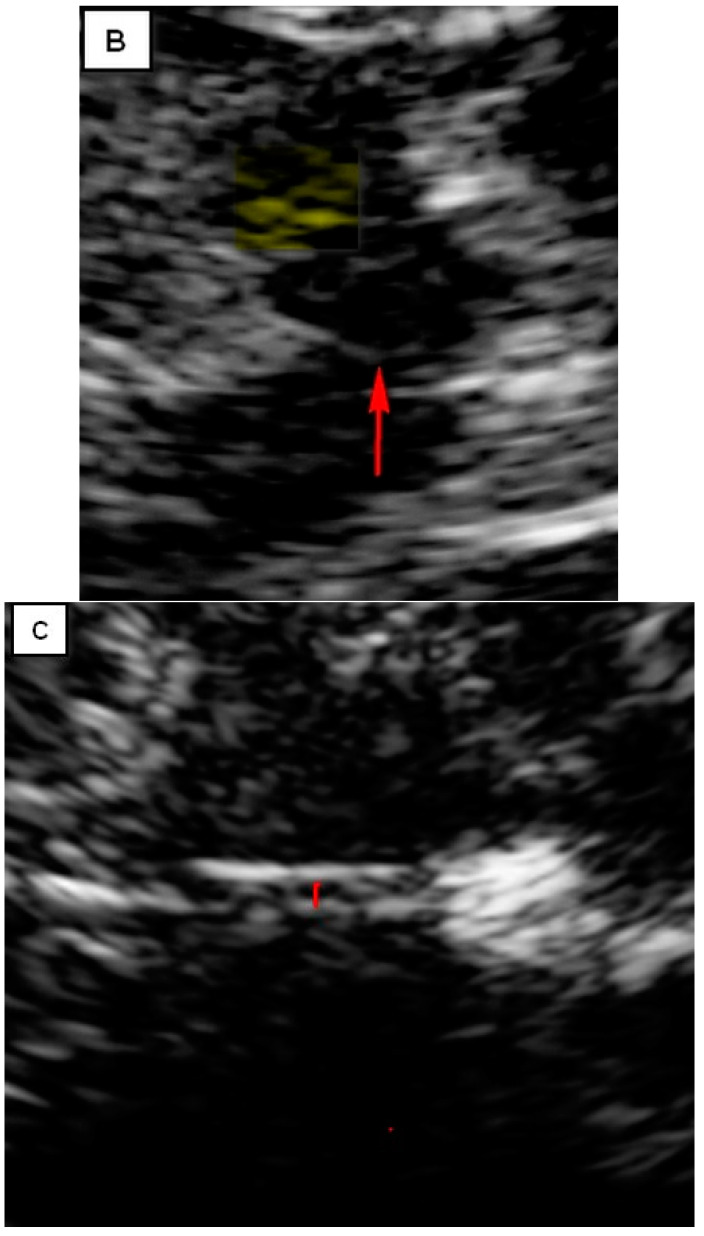

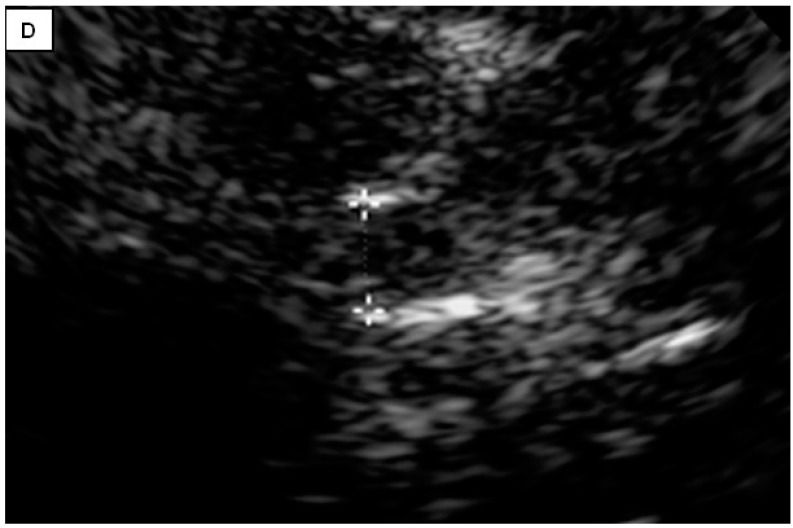
(**A**) Butterfly-shaped hypoechogenic mesencephalic brainstem insonation level. Normal echogenicity of the brainstem raphe (highly hyperechogenic continuous line, same echogenicity as red nucleus; red arrow); dotted like highly hyperechogenic red nucleus (NR) (yellow arrow); normal echogenicity of substantia nigra (SN) (white arrow). (**B**) Butterfly-shaped hypoechogenic mesencephalic brainstem insonation level. Hypoechogenic interrupted raphe (red arrow); hyperechogenic substantia nigra (SN) (blurred yellow area, above 0.19 cm^2^). (**C**) Third ventricle level insonation depicted as hyperechogenic parallel lines. Normal diameter of the third ventricle (inner borders marked with red line). (**D**) Third ventricle level insonation depicted as hyperechogenic parallel lines. Dilatated third ventricle (12 mm, inner borders marked with white asterisk).

TCS characteristics of interest in our study are illustrated in Table 2. The control subjects consisted of 31 healthy controls, of which there was a slight female predominance (54.8%). The mean age of control subjects was 45.7 ± 11.6 years. The brainstem raphe was discontinued in 33.8% of patients. The SN hyperechogenicity was identified in 79.7% of patients, which represented a significant pathological finding, Figure 1. Third ventricle enlargement (over 6 mm) was present in 79.7% of patients, which can be attributed to gross (whole) brain atrophy, Figure 1. The fourth ventricle was enlarged in 45.9% of patients, which can directly be attributed to the atrophy of the cerebellum and its reduction in volume. The hyperechogenicity of other brain structures, such as the NR, the lentiform nucleus (NL) and the dentate nucleus (ND), is also illustrated in Table 2. We found a statistically significant difference in the hyperechogenicity of SN on the left and the sum quantitively, as well as in the frequencies of hyperintensity between patients with degenerative ataxias and the control subjects (*p* < 0.05). We have also found statistical significance in the difference between these two groups in the domain of the III and IV ventricles (*p* < 0.01). Results of mesencephalic atrophy are also presented in Table 2. 

A positive and strong statistically significant correlation (*p* < 0.01) was found between dystonia and SN hyperechogenicity. Also, a positive and strong statistically significant correlation was found (*p* < 0.05) between rigidity and dyskinesia and SN hyperechogenicity. Such results are illustrated in Table 3. The higher SARA total score is statistically significantly correlated with the larger diameter of the III (r = 0.373; *p* = 0.001) and IV ventricles (r = 0.324; *p* = 0.005). Discontinued raphe was statistically significantly associated with Hamilton depression score (r = −0.225; *p* = 0.054). However, it must be said that it is a threshold value; that is, the level of significance is about 5%, so it can be interpreted that there is a negative and statistically significant correlation between the two mentioned variables. 

Table 4 shows the TCS characteristics of patients with neurodegenerative ataxias. Patients with SCA1, SCA2 and sporadic neurodegenerative ataxia were compared according to their TCS characteristics, and results have shown that between the different groups of patients, there is a difference in TCS characteristics, and no differences were found among the three groups (*p* > 0.05). Such results can be attributed to the small sample of patients in each group. However, some differences in frequencies could be observed, such as the higher frequency of discontinued raphe in the sporadic ataxia group compared to the SCA1 and SCA 2 groups. Also, the enlargement of the fourth ventricle was found to be the most frequent in the SCA1 group of patients compared to the SCA2 and sporadic group.

In Table 5. the TCS characteristics of patients with hereditary neurodegenerative ataxias are compared to each other. Patient groups with confirmed diagnoses of SCA1, SCA2 and FRDA were compared. A statistically significant difference (*p* < 0.05) was found between these compared groups in regard to SN hyperechogenicity. We have found a statistically significant difference in the echogenicity of SN left between these groups (*p* < 0.05).

When comparing TCS characteristics of sporadic neurodegenerative ataxias and MSAc, a statistically significant difference (*p* < 0.05) was found between these two groups in the domain of raphe continuity. The sporadic neurodegenerative ataxia group had more frequently continued (normal) brainstem raphe than the MSAc group, which might be attributed to the more frequent presence of depression in the MSAc group of subjects. Such results are shown in Table 6. 

## 4. Discussion

Our results have shown a statistically significant correlation between the hyperechogenicity of substantia nigra and extrapyramidal signs, precisely rigidity, dyskinesia and dystonia. Such results are supported by previously published results [23].

We have obtained results suggesting a statistically significant correlation between SN hyperechogenicity and dystonia, which is in agreement with previous studies. A study conducted on dopa-responsive dystonia patients showed similar results, suggesting that SN hyperechogenicity might correlate with the symptoms of dystonia [24]. 

In a previous study, we found the hyperechogenicity of SN in 67% of patients with SCA2, and since patients did have Parkinsonian signs, we concluded that hyperechogenicity might reflect early strionigral degeneration [12]. Parkinsonism as a dominant clinical phenotype has been described in patients with SCA2, and pathological findings frequently reveal marked degeneration in the SN beside severe neuronal loss in the pons, cerebellum, inferior olive and dorsal columns [25,26,27]. SCA3 is also characterized by ataxia and variable findings of parkinsonism, dystonia and peripheral neuropathy [28] and a marked hyperechogenicity of SN was reported in this group of patients [29]. 

Changes in the echogenicity of SN are a frequent finding in AD-SCA characterized by frequent involvement of the nigrostriatal pathways.

Our results have shown a strong and statistically significant positive correlation between the TCS-evaluated diameters of the third and fourth ventricles, which correspond to the SARA scores attributed to the severity of ataxia. In the literature, it is documented that in patients with spinocerebellar ataxia (SCA), especially SCA 2 and 3, alterations of ventricle size are present. Accordingly, SCA 2 has been associated with the enlargement of the third ventricle, while in SCA 3, patients that have larger diameters of the fourth ventricle have been observed on TCS images [30]. 

Moreover, we have compared different aspects of ataxia using the SARA scale, in particular, the truncal SARA scale, the speech SARA scale and the extremity SARA scale, and correlated them with the TCS diameters of the third and fourth ventricles. The results have shown a statistically significant correlation between all ventricle sizes and all SARA scores in all three domains, which is also supported by works of other investigators who had come to the same conclusion [12,30,31,32]. 

Research investigating TCS characteristics in patients with FRDA has shown that also in these patients, the diameter of the fourth and third ventricles, as well as of the lateral ventricles, showed enlargement on TCS; however, after correction to age, neither disease duration nor ICARS score correlated to the diameter of the ventricles [33]. Moreover, in FRDA, it has been observed that the mean sum area of SN echogenicity was significantly smaller in FRDA patients than in healthy controls and that SN echogenicity showed an inverse correlation with ataxia severity [33]. In our study investigating midbrain structures, we observed a higher frequency of ND in patients with FRDA. Such results are in agreement with data obtained by Synofsky et al., who found that 85% of patients with FRDA exhibited DN hyperechogenicity [34].

Because of the high comorbidity of psychiatric disorders in neurodegenerative diseases, we have evaluated the level of depression using HAMD and correlated it with raphe continuity, which has shown a borderline negative correlation between the severity of depression according to the scale and raphe continuity. Previous evaluations of TCS characteristics of raphe in depressive states of various etiology conclude that the prevalence of hypoechogenic nucleus raphe appears to be similar in isolated depressive disorders and in depression associated with degenerative brain diseases. This data can be used as a predictor of response to distinct antidepressant medication or suicidal ideation [35]. 

The main limitation of our study is represented by the lack of availability of concomitant MRI images from our study cohort to be used for correlation analysis with TCS data, an issue due to the retrospective nature of the study design.

## 5. Conclusions

In our study, we have shown the main TCS characteristics of patients with neurodegenerative ataxias. In such patients, the echogenicity of SN has been linked to extrapyramidal signs, and raphe discontinuity to depression. Furthermore, ataxia and its clinical subtypes have positively correlated with the IV ventricle diameter, indicating brain atrophy and brain mass reduction. Based on our results and previous literature data, TCS appears to be an effective, dependable and reproducible complementary imaging technique for monitoring patients with neurodegenerative ataxias. Longitudinal prospective studies evaluating in parallel clinical rating scores of disease severity, brain MRI and TCS findings are needed to definitively assess the value of TCS as a diagnostic tool able to monitor neurodegenerative changes occurring in degenerative ataxias.

## Figures and Tables

**Table 1:** Clinical and demographic profile of participants.

Variable	Value
Sex (%)	
Male	47 (63.5)
Female	27 (36.5)
Age (years)	48.1 ± 12.9 (range 22–69)
BMI (kg/m^2^)	23.5 ± 2.9 (range 16.4–31.4)
Age at onset (years)	36.5 ± 16.5 (range 0–68)
Disease duration (years)	11.7 ± 9.6 (range 1–41)
Time to walking support tool (years)	8.4 ± 6.7 (range 0.3–25)
Time to wheelchair (years)	12.0 ± 7.4 (range 0–25)
SARA total score	15.3 ± 6.8 (range 3–39)
CCFS total score	1.0 ± 0.2 (range 0–1.78)
SCAFI total score	−0.1 ± 0.7 (range −2.0–2.0)
INAS total score	4.5 ± 2.0 (range 1–9)
SCOPA AUT—total score	5.1 ± 6.8 (range 0–34)
ACE—R total score	77.7 ± 17.2 (range 0–100)
MMSE	25.4 ± 4.5 (range 9–30)
HAMD	8.0 ± 6.1 (range 0–23)
Disease (%)	
AD	27 (36.5)
AR	15 (20.3)
Sporadic	25 (33.8)
MSAc	7 (9.5)

BMI—Body mass index, Scale for Assessment and Rating of Ataxia (SARA), Spinocerebellar Ataxia Functional Index (SCAFI), Composite Cerebellar Functional Severity Score (CCFS), Inventory of Non-Ataxia Signs (INAS), Autonomic Symptoms Assessment Questionnaire (SCOPA AUT) Mini-Mental State Examination (MMSE), Addenbrooke’s Cognitive Examination—Revised version (ACE-R), Hamilton Depression Rating Scale (HAMD), autosomal dominant (AD), autosomal recessive (AR), cerebellar type of multiple system atrophy (MSAc).

**Table 2:** TCS profile of patients with neurodegenerative cerebellar ataxias.

Variable	Patients	Control Subjects	*p*
Raphe (%) Discontinued Continued	25 (33.8)	4 (12.9)	0.029 *
	49 (66.2)	27 (87.1)	
SN Left	0.12 ± 0.05	0.10 ± 0.07	0.174
	(range 0.00–0.34)	(range 0.00–0.20)	
SN Right	0.12 ± 0.05	0.09 ± 0.07	**0.048**
	(range 0.01–0.29)	(range 0.00–0.28)	
SN sum	0.24 ± 0.08	0.20 ± 0.11	**0.045**
	(range 0.9–0.56)	(range 0.00–0.42)	
Sum SN (%)			**<0.001**
Normal	15 (20.3)	16 (51.6)	
Hyperechogenic	59 (79.7)	15 (48.4)	
SN (%)			0.101
Until 0.18	15 (20.3)	16	
0.19–0.24	30 (40.5)	14	
Over 0.24	29 (39.2)	1	
III ventricle diameter	7.0 ± 0.2 (range 3–13)	5.5 ± 2.3 (range 3–11.2)	**0.001**
III ventricle (%)			**<0.001**
Normal	14 (18.9)	26	
Enlarged 6 mm	59 (79.7)	5	
IV ventricle diameter	5.52 ± 0.2 (range 2–12)	–	
IV ventricle (%)			**0.009**
Normal	40 (54.1)	29 (93.5)	
Enlarged	34 (45.9)	2 (6.5)	
Hyperechogenicity of other nuclei (%)			0.173
Hyperechogenicity NR	14 (18.9)	4 (12.9)	
Hyperechogenicity ND	4 (5.4)	0 (0)	
Hyperechogenicity NL	2 (2.7)	3 (9.7)	
Mesencephalic atrophy	5 (6.8)	1 (3.2)	

SN substantia nigra, NR red nucleus, ND dentate nucleus, NL lentiform nucleus. Bold values denote statistical significance * *p* < 0.05.

**Table 3:** Correlation between extrapyramidal signs and TCS characteristics of substantia nigra.

	Rigidity	Dyskinesia	Dystonia
SN left	0.137	−0.149	0.007
SN right	0.095	−0.118	−0.167
SN sum	0.154	−0.025	−0.012
Hyperechogenic SN	0.229 *	0.229 *	0.309 **

SN substantia nigra. Bold values denote statistical significance * *p* < 0.05, ** *p* < 0.01.

**Table 4:** TCS profile of patients with SCA1, SCA2 and sporadic neurodegenerative ataxia.

Variable	SCA1	SCA2	Sporadic	*p*-Value
Raphe (%)				0.491
Discontinued	2 (20.0)	1 (16.7)	9 (36.0)	
Continued	8 (80.0)	5 (83.3)	16 (64.0)	
SN Left	0.10 ± 0.02	0.13 ± 0.05	0.13 ± 0.06	0.342
	(range 0.05–0.14)	(range 0.08–0.19)	(range 0.04–0.34)	
SN Right	0.11 ± 0.04	0.11 ± 0.07	0.12 ± 0.06	0.870
	(range 0.04–0.17)	(range 0.02–0.23)	(range 0.01–0.23)	
SN sum	0.20 ± 0.06	0.24 ± 0.09	0.24 ± 0.11	0.599
	(range 0.9–0.29)	(range 0.16–0.41)	(range 0.11–0.56)	
Sum SN (%)				0.143
Normal	10 (100.0)	4 (66.7)	22 (88.0)	
Hyperechogenic	0 (0.0)	2 (33.3)	3 (12.0)	
SN (%)				0.526
Until 0.18	5 (50.0)	2 (33.3)	6 (24.0)	
0.19–0.24	2 (20.0)	3 (50.0)	11 (44.0)	
Over 0.24	3 (30.0)	1 (16.7)	8 (32.0)	
III ventricle diameter	6.7 ± 1.8	6.7 ± 2.5	6.5 ± 2.2	0.970
	(range 4–10)	(range 4–11)	(range 3–13)	
III ventricle (%) Normal Enlarged 6 mm				0.823
	8 (80.0)	5 (83.3)	22 (88.0)	
	2 (20.0)	1 (16.7)	3 (12.0)	
IV ventricle diameter	6.2 ± 1.8	5.8 ± 2.3	5.4 ± 2.2	0.600
	(range 4–9)	(range 4–10)	(range 3–10)	
IV ventricle (%)				0.508
Normal	5 (55.6)	4 (66.7)	19 (76.0)	
Enlarged	4 (44.4)	2 (33.3)	6 (24.0)	
Hiyperechogenicity of other nuclei (%)				0.915
Hyperechogenicity NR	2 (22.2)	1 (16.7)	6 (24.0)	
Hyperechogenicity NL	0 (0.0)	0 (0.0)	1 (0.04)	
Mesencephalic atrophy	1 (11.1)	0 (0.0)	2 (0.08)	

SN substantia nigra, NR red nucleus, ND dentate nucleus, NL lentiform nucleus.

**Table 5:** TCS profile of patients with neurodegenerative cerebellar ataxias. Comparation between TCS characteristics of hereditary ataxias is shown.

Variable	SCA1	SCA2	FRDA	*p*-Value
Raphe (%)				0.569
Discontinued	2 (20.0)	1 (16.7)	0 (0.0)	
Continued	8 (80.0)	5 (83.3)	5 (100.0)	
SN Left	0.10 ± 0.02	0.13 ± 0.05	0.16 ± 0.05	0.022 *
	(range 0.05–0.14)	(range 0.08–0.19)	(range 0.10–0.22)	
SN Right	0.11 ± 0.04	0.11 ± 0.07	0.11 ± 0.02	0.996
	(range 0.04–0.17)	(range 0.02–0.23)	(range 0.10–0.14)	
SN sum	0.20 ± 0.06	0.24 ± 0.09	0.27 ± 0.06	0.267
	(range 0.9–0.29)	(range 0.16–0.41)	(range 0.20–0.36)	
Sum SN (%)				0.102
Normal	10 (100.0)	4 (66.7)	3 (60.0)	
Hyperechogenic	0 (0.0)	2 (33.3)	2 (40.0)	
SN (%)				0.267
Until 0.18	5 (50.0)	2 (33.3)	0 (0.0)	
0.19–0.24	2 (20.0)	3 (50.0)	2 (40.0)	
Over 0.24	3 (30.0)	1 (16.7)	3 (60.0)	
III ventricle diameter	6.7 ± 1.8	6.7 ± 2.5	9.2 ± 2.0	0.090
	(range 4–10)	(range 4–11)	(range 7–11)	
III ventricle (%)				0.202
Normal	8 (80.0)	5 (83.3)	2 (40.0)	
Enlarged 6 mm	2 (20.0)	1 (16.7)	3 (60.0)	
IV ventricle diameter	6.2 ± 1.8	5.8 ± 2.3	6.0 ± 2.3	0.938
	(range 4–9)	(range 4–10)	(range 4–9)	
IV ventricle (%)				0.912
Normal	5 (55.6)	4 (66.7)	3 (60.0)	
Enlarged	4 (44.4)	2 (33.3)	2 (40.0)	
Hiyperechogenicity of other nuclei (%)				0.064
Hyperechogenicity NR	2 (22.2)	1 (16.7)	0 (0.0)	
Hyperechogenicity ND	0 (0.0)	0 (0.0)	4 (80.0)	
Hyperechogenicity NL	0 (0.0)	0 (0.0)	0 (0.0)	
Mesencephalic atrophy	1 (11.1)	0 (0.0)	0 (0.0)	

SN substantia nigra, NR red nucleus, ND dentate nucleus, NL lentiform nucleus. Bold values denote statistical significance * *p* < 0.05.

**Table 6:** TCS profile of patients with neurodegenerative cerebellar ataxias showing comparison of TCS characteristics between sporadic neurodegenerative ataxias and MSAc.

Variable	Sporadic	MSAc	*p*-Value
Raphe (%)			0.036 *
Discontinued	9 (36.0)	5 (83.3)	
Continued	16 (64.0)	1 (16.7)	
SN Left	0.13 ± 0.06	0.18 ± 0.16	0.166
	(range 0.04–0.34)	(range 0.10–0.50)	
SN Right	0.12 ± 0.06	0.14 ± 0.08	0.408
	(range 0.01–0.23)	(range 0.05–0.29)	
SN sum	0.24 ± 0.11	0.25 ± 0.10	0.956
	(range 0.11–0.56)	(range 0.10–0.41)	
Sum SN (%)			0.759
Normal	22 (88.0)	5 (83.3)	
Hyperechogenic	3 (12.0)	1 (16.7)	
SN (%)			0.709
Until 0.18	6 (24.0)	1 (16.7)	
0.19–0.24	11 (44.0)	2 (33.3)	
Over 0.24	8 (32.0)	3 (50.0)	
III ventricle diameter	6.5 ± 2.2	7.7 ± 0.8	0.220
	(range 3–13)	(range 7–9)	
III ventricle (%)			0.221
Normal	5 (20.8)	0 (0.0)	
Enlarged 6 mm	19 (79.2)	6 (100.0)	
IV ventricle diameter	5.4 ± 2.2	4.8 ± 1.6	0.559
	(range 3–10)	(range 3–7)	
IV ventricle (%)			0.639
Normal	19 (76.0)	4 (66.7)	
Enlarged	6 (24.0)	2 (33.3)	
Hiyperechogenicity of other nuclei (%)			/
Hyperechogenicity NR	6 (24.0)	0 (0.0)	
Hyperechogenicity ND	0 (0.0)	0 (0.0)	
Hyperechogenicity NL	1 (4.0)	0 (0.0)	
Mesencephalic atrophy	2 (8.0)	0 (0.0)	

SN substantia nigra, NR red nucleus, ND dentate nucleus, NL lentiform nucleus. Bold values denote statistical significance * *p* < 0.05.

## Data Availability

The data from this study are available on request from the corresponding author. The data are not publicly available due to privacy and ethical restrictions.
